# Ohio Medical Convention

**Published:** 1847-11

**Authors:** 


### OHIO MEDICAL CONVENTION.

The proceedings of the Medical Convention of Ohio, held at Columbus, in May last, have been issued in a pamphlet of fifty-nine pages. Among these are several papers of interest read by members on various professional subjects. Our friend and valued correspondent, Dr. Dawson, of Jamestown, is the author of one on *puerperal fever*, from which we propose making some extracts in our next number. The style in which this pamphlet has been executed by the printers is not worthy of the matter contained in it. Typographical errors of the most glaring kind strike one on almost every page.

